# Crystal structure of phen­yl(pyridin-2-yl)methanol

**DOI:** 10.1107/S1600536814016857

**Published:** 2014-08-01

**Authors:** Haneol Kim, Sung Kwon Kang

**Affiliations:** a Department of Chemistry, Chungnam National University, Daejeon 305-764, Republic of Korea

**Keywords:** crystal structure, phen­yl(pyridin-2-yl)methanol, hydrogen bonding

## Abstract

In the title compound, C_12_H_11_NO, the pyridine and phenyl rings are inclined to each other by 71.42 (10)°. In the crystal, O—H⋯N hydrogen bonds link the mol­ecules into helical chains extending along the *c*-axis direction.

## Related literature   

For the synthesis of the title compound and some derivatives, see: Frassoldati *et al.* (2013 ▶); Tao *et al.* (2012 ▶). For its use in synthesis, see: Miyamura *et al.* (2008 ▶); Lucchesi *et al.* (2008 ▶); Lash *et al.* (2007 ▶); Szajna *et al.* (2004 ▶).
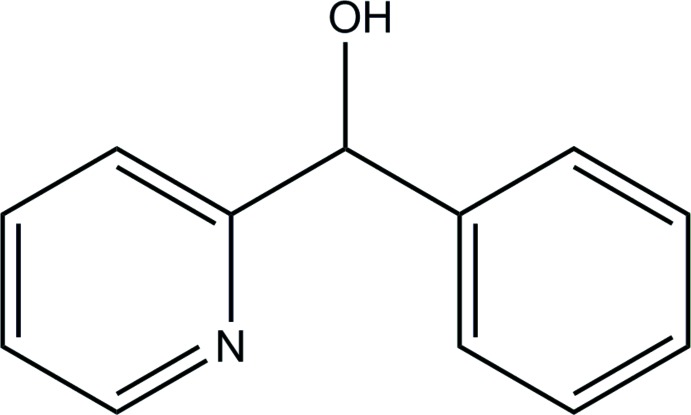


## Experimental   

### Crystal data  

C_12_H_11_NO
*M* *_r_* = 185.22
Orthorhombic, 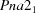

*a* = 7.4385 (8) Å
*b* = 14.3429 (16) Å
*c* = 9.2255 (10) Å
*V* = 984.27 (19) Å^3^
*Z* = 4
Mo *K*α radiation
μ = 0.08 mm^−1^
*T* = 296 K
0.3 × 0.26 × 0.18 mm

### Data collection  

Bruker SMART CCD area-detector diffractometer
7290 measured reflections
2245 independent reflections
1190 reflections with *I* > 2σ(*I*)
*R*_int_ = 0.055

### Refinement  

*R*[*F* ^2^ > 2σ(*F* ^2^)] = 0.039
*wR*(*F* ^2^) = 0.084
*S* = 0.81
2245 reflections
131 parameters
1 restraint
H atoms treated by a mixture of independent and constrained refinement
Δρ_max_ = 0.09 e Å^−3^
Δρ_min_ = −0.12 e Å^−3^

### 

Data collection: *SMART* (Bruker, 2002 ▶); cell refinement: *SAINT* (Bruker, 2002 ▶); data reduction: *SAINT*; program(s) used to solve structure: *SHELXS2013* (Sheldrick, 2008 ▶); program(s) used to refine structure: *SHELXL2013* (Sheldrick, 2008 ▶); molecular graphics: *ORTEP-3 for Windows* (Farrugia, 2012 ▶); software used to prepare material for publication: *WinGX* (Farrugia, 2012 ▶).

## Supplementary Material

Crystal structure: contains datablock(s) global, I. DOI: 10.1107/S1600536814016857/su2760sup1.cif

Structure factors: contains datablock(s) I. DOI: 10.1107/S1600536814016857/su2760Isup2.hkl

Supporting information file. DOI: 10.1107/S1600536814016857/su2760Isup3.cml

**Fig. 1.**
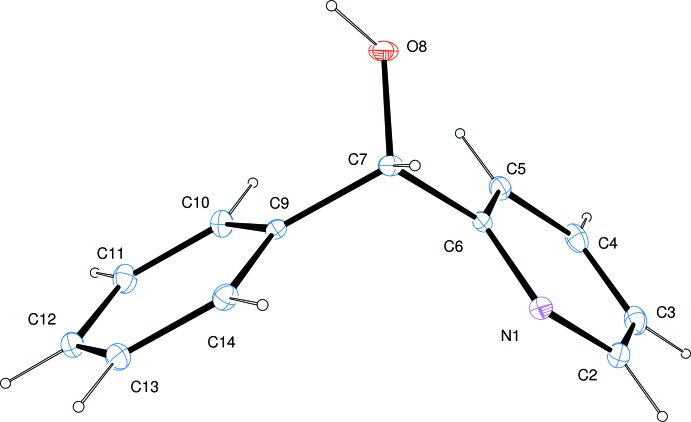
Molecular structure of the title molecule, with atom labelling. The displacement ellipsoids are drawn at the 30% probability level.

. DOI: 10.1107/S1600536814016857/su2760fig1.tif
Mol­ecular structure of the title mol­ecule, with atom labelling. The displacement ellipsoids are drawn at the 30% probability level.

**Fig. 2.**
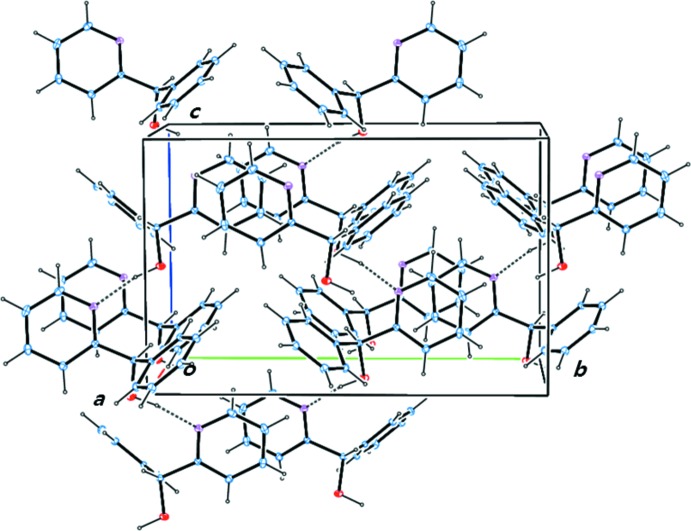
A view along the a axis of the crystal packing of the title compound, showing molecules linked by O—H···N hydrogen bonds (dashed lines; see Table 1 for details).

. DOI: 10.1107/S1600536814016857/su2760fig2.tif
A view along the a axis of the crystal packing of the title compound, showing mol­ecules linked by O—H⋯N hydrogen bonds (dashed lines; see Table 1 for details).

CCDC reference: 1015307

Additional supporting information:  crystallographic information; 3D view; checkCIF report

## Figures and Tables

**Table 1:** Hydrogen-bond geometry (Å, °)

*D*—H⋯*A*	*D*—H	H⋯*A*	*D*⋯*A*	*D*—H⋯*A*
O8—H8⋯N1^i^	0.98 (5)	1.85 (5)	2.809 (4)	166 (4)

Symmetry code: (i) 
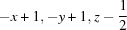
.

## supplementary crystallographic information

### S1. Experimental

To a solution of 2-benzoylpyridine (5.0 g, 0.027 mol) in EtOH (60 ml) was added NaBH_4_ (3.13 g, 0.083 mol) slowly at room temperature. The solution was stirred gently for 1 h. After adding 60 ml H_2_O, this solution was heated at 363 K for 15 min. After cooling, the product was extracted with AcOEt (50 ml). The solvent was evaporated under reduced pressure to leave a pale green oil. Colourless crystals of the title compound were obtained by slow evaporation of a solution in EtOH at room temperature.

### S2. Refinement

Atom H8 of the OH group was located in a difference Fourier map and freely refined. C-bound H atoms were positioned geometrically and refined using a riding model: C—H = 0.93 - 0.98 Å with *U*_iso_(H) = 1.2*U*_eq_(C).

### Crystal data

**Table:**

C_12_H_11_NO	*F*(000) = 392
*M**_r_* = 185.22	*D*_x_ = 1.25 Mg m^−^^3^
Orthorhombic, *P**n**a*2_1_	Mo *K*α radiation, λ = 0.71073 Å
Hall symbol: P 2c -2n	Cell parameters from 973 reflections
*a* = 7.4385 (8) Å	θ = 2.6–19.7°
*b* = 14.3429 (16) Å	µ = 0.08 mm^−^^1^
*c* = 9.2255 (10) Å	*T* = 296 K
*V* = 984.27 (19) Å^3^	Block, colourless
*Z* = 4	0.3 × 0.26 × 0.18 mm

### Data collection

**Table:**

Bruker SMART CCD area-detector diffractometer	*R*_int_ = 0.055
Radiation source: fine-focus sealed tube	θ_max_ = 27.5°, θ_min_ = 2.6°
φ and ω scans	*h* = −7→9
7290 measured reflections	*k* = −18→18
2245 independent reflections	*l* = −11→11
1190 reflections with *I* > 2σ(*I*)	

### Refinement

**Table:**

Refinement on *F*^2^	1 restraint
Least-squares matrix: full	Hydrogen site location: mixed
*R*[*F*^2^ > 2σ(*F*^2^)] = 0.039	H atoms treated by a mixture of independent and constrained refinement
*wR*(*F*^2^) = 0.084	*w* = 1/[σ^2^(*F*_o_^2^) + (0.0303*P*)^2^] where *P* = (*F*_o_^2^ + 2*F*_c_^2^)/3
*S* = 0.81	(Δ/σ)_max_ < 0.001
2245 reflections	Δρ_max_ = 0.09 e Å^−^^3^
131 parameters	Δρ_min_ = −0.12 e Å^−^^3^

### Special details

**Table:**

Geometry. All e.s.d.'s (except the e.s.d. in the dihedral angle between two l.s. planes) are estimated using the full covariance matrix. The cell e.s.d.'s are taken into account individually in the estimation of e.s.d.'s in distances, angles and torsion angles; correlations between e.s.d.'s in cell parameters are only used when they are defined by crystal symmetry. An approximate (isotropic) treatment of cell e.s.d.'s is used for estimating e.s.d.'s involving l.s. planes.

### Fractional atomic coordinates and isotropic or equivalent isotropic displacement parameters (Å^2^)

**Table:**

	*x*	*y*	*z*	*U*_iso_*/*U*_eq_	
N1	0.4374 (3)	0.37463 (19)	0.8538 (3)	0.0544 (7)	
C2	0.4309 (5)	0.2902 (3)	0.9150 (5)	0.0738 (12)	
H2	0.4616	0.2851	1.0124	0.089*	
C3	0.3819 (5)	0.2112 (3)	0.8433 (6)	0.0773 (12)	
H3	0.3794	0.154	0.8907	0.093*	
C4	0.3365 (4)	0.2177 (2)	0.7004 (5)	0.0733 (12)	
H4	0.302	0.1651	0.6485	0.088*	
C5	0.3426 (4)	0.3039 (2)	0.6344 (4)	0.0582 (9)	
H5	0.3119	0.3104	0.5372	0.07*	
C6	0.3949 (4)	0.3804 (2)	0.7141 (3)	0.0434 (7)	
C7	0.4046 (4)	0.4769 (2)	0.6482 (3)	0.0501 (8)	
H7	0.5021	0.5116	0.6949	0.06*	
O8	0.4471 (3)	0.46457 (19)	0.5006 (3)	0.0700 (7)	
H8	0.474 (5)	0.526 (3)	0.460 (5)	0.111 (16)*	
C9	0.2299 (3)	0.52994 (18)	0.6693 (3)	0.0409 (7)	
C10	0.0771 (4)	0.5047 (2)	0.5952 (4)	0.0587 (9)	
H10	0.0817	0.4549	0.5307	0.07*	
C11	−0.0819 (4)	0.5515 (3)	0.6146 (4)	0.0715 (11)	
H11	−0.1837	0.5334	0.5633	0.086*	
C12	−0.0908 (5)	0.6244 (3)	0.7087 (4)	0.0679 (10)	
H12	−0.1989	0.6557	0.7224	0.081*	
C13	0.0579 (5)	0.6514 (2)	0.7824 (4)	0.0697 (10)	
H13	0.0515	0.7017	0.8459	0.084*	
C14	0.2204 (4)	0.6043 (2)	0.7639 (4)	0.0570 (8)	
H14	0.3219	0.623	0.8151	0.068*	

### Atomic displacement parameters (Å^2^)

**Table:**

	*U*^11^	*U*^22^	*U*^33^	*U*^12^	*U*^13^	*U*^23^
N1	0.0530 (17)	0.061 (2)	0.0488 (19)	0.0047 (13)	0.0014 (14)	0.0027 (15)
C2	0.066 (3)	0.084 (3)	0.071 (3)	0.019 (2)	0.008 (2)	0.024 (2)
C3	0.059 (2)	0.059 (3)	0.114 (4)	0.006 (2)	0.015 (2)	0.029 (3)
C4	0.059 (2)	0.049 (2)	0.112 (4)	0.0045 (17)	0.006 (3)	−0.008 (2)
C5	0.056 (2)	0.057 (2)	0.062 (2)	0.0079 (16)	0.0005 (17)	−0.0047 (19)
C6	0.0362 (15)	0.0463 (19)	0.0476 (19)	0.0058 (13)	0.0045 (15)	−0.0004 (15)
C7	0.0500 (18)	0.0578 (19)	0.0426 (19)	−0.0030 (15)	0.0052 (15)	−0.0001 (16)
O8	0.0827 (17)	0.0741 (18)	0.0530 (15)	0.0020 (14)	0.0260 (13)	0.0028 (13)
C9	0.0452 (16)	0.0391 (15)	0.0383 (15)	−0.0036 (14)	0.0017 (14)	0.0062 (14)
C10	0.059 (2)	0.047 (2)	0.069 (2)	−0.0019 (17)	−0.0103 (18)	−0.0040 (17)
C11	0.052 (2)	0.072 (2)	0.090 (3)	0.0003 (19)	−0.009 (2)	0.010 (2)
C12	0.061 (2)	0.071 (2)	0.072 (3)	0.016 (2)	0.011 (2)	0.014 (2)
C13	0.091 (3)	0.057 (2)	0.061 (2)	0.018 (2)	0.004 (2)	−0.005 (2)
C14	0.066 (2)	0.0535 (18)	0.0512 (19)	−0.0022 (16)	−0.0088 (18)	−0.0012 (17)

### Geometric parameters (Å, º)

**Table:**

N1—C6	1.329 (4)	C7—H7	0.98
N1—C2	1.338 (4)	O8—H8	0.98 (5)
C2—C3	1.362 (5)	C9—C10	1.374 (4)
C2—H2	0.93	C9—C14	1.380 (4)
C3—C4	1.364 (5)	C10—C11	1.372 (4)
C3—H3	0.93	C10—H10	0.93
C4—C5	1.379 (5)	C11—C12	1.361 (5)
C4—H4	0.93	C11—H11	0.93
C5—C6	1.376 (4)	C12—C13	1.354 (5)
C5—H5	0.93	C12—H12	0.93
C6—C7	1.514 (4)	C13—C14	1.395 (4)
C7—O8	1.408 (4)	C13—H13	0.93
C7—C9	1.518 (4)	C14—H14	0.93
			
C6—N1—C2	117.2 (3)	C9—C7—H7	108.8
N1—C2—C3	123.9 (4)	C7—O8—H8	108 (3)
N1—C2—H2	118.1	C10—C9—C14	118.4 (3)
C3—C2—H2	118.1	C10—C9—C7	120.8 (3)
C2—C3—C4	118.6 (4)	C14—C9—C7	120.8 (3)
C2—C3—H3	120.7	C11—C10—C9	121.3 (3)
C4—C3—H3	120.7	C11—C10—H10	119.4
C3—C4—C5	118.7 (4)	C9—C10—H10	119.4
C3—C4—H4	120.7	C12—C11—C10	120.1 (4)
C5—C4—H4	120.7	C12—C11—H11	120
C6—C5—C4	119.2 (3)	C10—C11—H11	120
C6—C5—H5	120.4	C13—C12—C11	120.0 (3)
C4—C5—H5	120.4	C13—C12—H12	120
N1—C6—C5	122.4 (3)	C11—C12—H12	120
N1—C6—C7	115.8 (3)	C12—C13—C14	120.5 (3)
C5—C6—C7	121.8 (3)	C12—C13—H13	119.8
O8—C7—C6	106.5 (3)	C14—C13—H13	119.8
O8—C7—C9	112.3 (2)	C9—C14—C13	119.8 (3)
C6—C7—C9	111.4 (2)	C9—C14—H14	120.1
O8—C7—H7	108.8	C13—C14—H14	120.1
C6—C7—H7	108.8		

### Hydrogen-bond geometry (Å, º)

**Table:**

*D*—H···*A*	*D*—H	H···*A*	*D*···*A*	*D*—H···*A*
O8—H8···N1^i^	0.98 (5)	1.85 (5)	2.809 (4)	166 (4)

Symmetry code: (i) −*x*+1, −*y*+1, *z*−1/2.
