# Supplementary figures and images for: Crystal structure of phen­yl(pyridin-2-yl)methanol
Source: Acta Crystallogr Sect E Struct Rep Online. 2014 Aug 1;70(Pt 9):o947. doi: 10.1107/S1600536814016857 (PMC4186067; doi:10.1107/S1600536814016857)

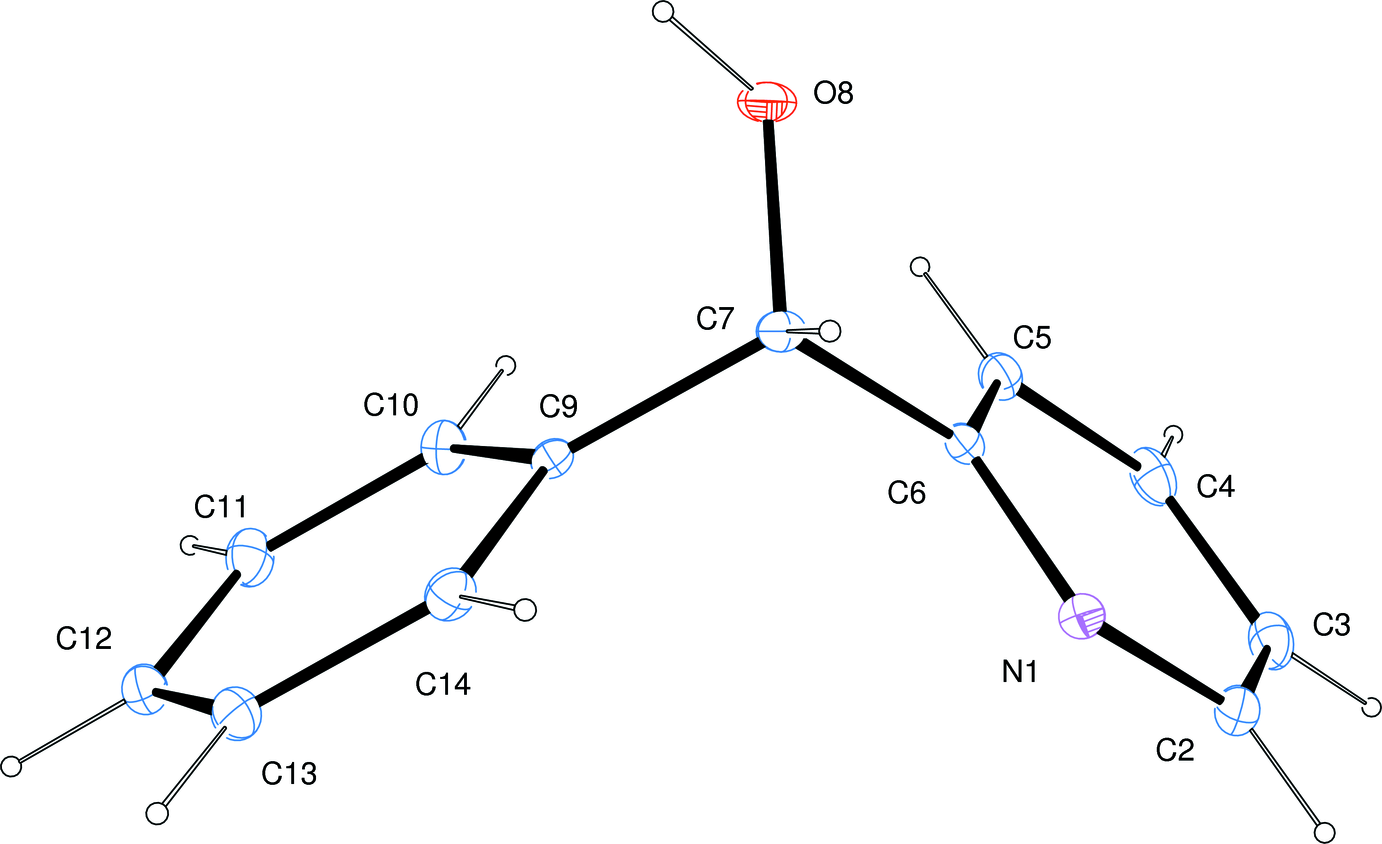

Supplement: Supplementary file 4 [file e-70-0o947-fig1.tif]

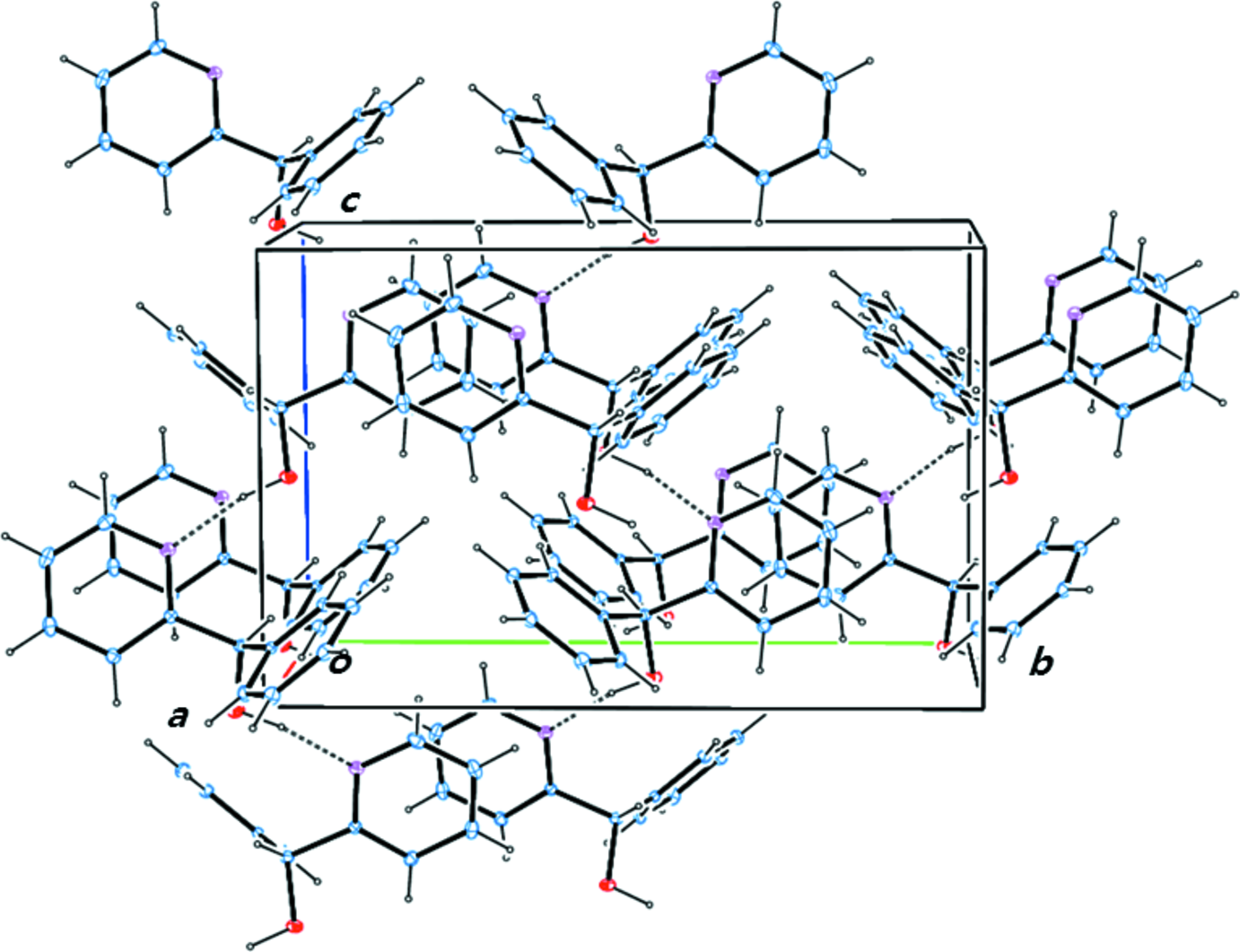

Supplement: Supplementary file 5 [file e-70-0o947-fig2.tif]
